# Editorial: Neurostimulation: exploring perceptual & cognitive enhancement

**DOI:** 10.3389/fpsyg.2025.1583115

**Published:** 2025-06-13

**Authors:** Adam J. Toth, Adam M. Bruton, Mark J. Campbell

**Affiliations:** ^1^Department of Physical Education and Sport Science, Health Research Institute, University of Limerick, Limerick, Ireland; ^2^Lero, The Research Ireland Centre for Software Research, University of Limerick, Limerick, Ireland; ^3^Centre for Sport Leadership, Stellenbosch University, Stellenbosch, South Africa; ^4^Department of Sport, Health and Exercise Sciences, Centre for Physical Activity in Health and Disease, Brunel University of London, London, United Kingdom; ^5^Centre for Integrated Research in Life and Health Sciences, University of Roehampton, London, United Kingdom

**Keywords:** neuromodulation, brain stimulation, tES, tDCS, TMS, transcranial electric stimulation

## Introduction

The use of electricity to artificially stimulate the nervous system dates to 15AD where electric fish were used to treat pain and headaches (Gildenberg, 2006). It wasn't until the 1960′s that modern neuromodulation emerged by way of deep brain, and then spinal cord, stimulation. Today, various forms of neurostimulation exist which involve stimulating peripheral sensory nerves (Toth et al., 2019; Maurer et al., 2001; Johnson and Wilson, 2018), the cortex (Toth et al., 2023; Galvin et al., 2023; Bruton et al., 2020), and the cerebellum (Lam et al., 2017), with both electric (tES) and magnetic fields (TMS). Our understanding of the mechanisms by which this stimulation affects neural communication has deepened and allowed for the controlled excitation and inhibition of cortical regions to probe and alter their function during various tasks (Castelli et al., 2025) Due to the ease with which motor performance can be evaluated, the primary motor cortex has been a popular target among neurostimulation studies, and, as such, promising results have been found regarding the efficacy of neurostimulation to influence and even augment various motor skills.

It is only more recently that neurostimulation has emerged as a promising avenue for enhancing cognitive and perceptual abilities. Transcranial electric current stimulation (tES), in particular, has garnered significant research attention given its simplicity and the fact that commercial tES devices are now readily available (Wexler, 2020). Given commercial devices can fall between regulatory gaps (clinical, consumer applications) some caution is needed especially regarding improperly applied neurostimulation (e.g., montage placement, modified devices and electric currents, contraindication screening). Although, transcranial magnetic stimulation (TMS) and transcranial pulse stimulation (TPS) have also been explored for their potential to improve attention, memory, reaction times, decision-making, and learning (Grafman and Wassermann, 1998). These techniques are being investigated across various domains, from clinical interventions for neurological conditions (Edwards et al., 2017) to cognitive enhancement in healthy individuals (Curtin et al., 2019). This editorial synthesizes recent findings pertaining to the implications for neurostimulation to enhance perceptual and cognitive abilities.

## Neurostimulation and cognition

Memory and learning are key areas where neurostimulation is being applied. A study combining computerized cognitive training (CCT) with different forms of transcranial electric stimulation (tDCS and tACS) found that stimulation effects varied based on individual factors such as age and education (Krebs et al.). Specifically, older individuals with higher education levels benefited more from tDCS, while younger individuals with less education responded better to tACS. Overall, this study highlights the importance of individualized stimulation protocols to maximize cognitive benefits. However, the findings regarding the efficacy for tES to enhance cognition in older populations is particularly interesting given it bolsters previous work (Kraft and Hampstead, 2024), as well as work using alterative neurostimulation techniques. For example, in another study (Zhang et al.) repetitive transcranial magnetic stimulation (rTMS) was examined as a potential intervention for early cognitive decline in individuals with subjective cognitive decline (SCD). Participants who received rTMS over the left dorsolateral prefrontal cortex (DLPFC) showed improvements in episodic memory, suggesting that neurostimulation may serve as an early intervention for Alzheimer's disease and related disorders. Furthermore, the study highlighted the role of long-term potentiation (LTP)-like cortical plasticity as a potential biomarker for cognitive improvement, paving the way for future research on neurostimulation in neurodegenerative conditions. Additionally, Zhang et al. evidenced LTP-like cortical plasticity behaviorally but also via TMS-EEG indices (TMS evoked potentials) and in doing so provide a more direct measure of cortical activation/reactivity following their intervention. This is in contrast to other work which tends to infer cellular mechanisms rather than direct receptor level measurement.

When considering more complex executive functions, such as problem-solving and cognitive flexibility, the effects of tES are less conclusive. A study investigating tDCS over the dorsolateral prefrontal cortex found that its efficacy in enhancing cognitive function was not as pronounced as its effects on motor learning (Toth et al.). Interestingly, the study suggested that sex differences might play a role in determining stimulation outcomes, with males showing greater benefits on simple attention tasks. This corroborates findings from a study using transcranial magnetic stimulation (TMS) to investigate how modulating activity in the right pre-supplementary motor area (RpSMA) and medial cerebellar vermis (MCV6) affected reaction times (Zhao et al.). The authors found that excitatory stimulation of the RpSMA and inhibitory stimulation of the MCV6 enhanced reaction speed in simple tasks but did not significantly impact more complex cognitive tasks. Furthermore, a systematic review by Wu et al. examined the effects of tACS on working memory, learning ability, and decision-making. The findings suggested that tACS enhances cognitive performance in athletes and healthy individuals, with effectiveness dependent on stimulation frequency, phase, area, and dose, highlighting the need for further research into individual differences in neurostimulation efficacy.

Semantic cognition, or the ability to process and retrieve meaningful information, has also been explored in neurostimulation research. A meta-analysis investigating whether TMS could simulate deficits in semantic control by targeting key brain regions involved in semantic retrieval found no significant effects of TMS after correcting for publication bias via a suite of methods (Funnel plots, Trim and Fill, Eggers Regression and Rank Correlation test, Selection Models, and Z curve analysis), suggesting that TMS may not be as effective for disrupting semantic processing as previously assumed (Ambrosini et al.). This calls for methodological improvements in future studies, including consideration of stimulation intensity, waveform and entrainment to underlying neural activity to enhance the reliability of findings in this area.

## Psychological effects of neurostimulation

Researchers have also explored whether neurostimulation can modulate cognitive dissonance, the well-documented psychological discomfort experienced when making difficult choices. A study using tDCS to inhibit the posterior medial frontal cortex (pMFC) found reduced preference changes in rejected options, while excitatory anodal stimulation showed no significant effect (Rybina et al.). These results highlight the potential of neurostimulation to influence decision-making processes, although the exact mechanisms require further investigation.

Finally, neurostimulation has also been explored in addressing attention deficits, particularly in individuals with conditions like attention-deficit/hyperactivity disorder (ADHD). A randomized controlled trial by Cheung et al. investigated the efficacy of transcranial pulse stimulation (TPS) for treating ADHD in young adolescents. The study demonstrated that TPS led to a 30% reduction in ADHD symptoms, with sustained benefits up to 3 months post-intervention. Despite not reporting standardized effect sizes which would greatly assist in the methodological and statistical reporting clarity the work highlights the utility of various forms of neurostimulation for not only cognitive enhancement, but as pharmacological interventions as well. Future work should examine the longer term benefits (e.g., 6–12 months) and or the requirement for maintenance neurostimulation sessions.

## Future applications for neurostimulation

Beyond traditional cognitive tasks, neurostimulation is being applied in realistic virtual environments to study its effects on human behavior. A review on tDCS in driving and flight simulators found that stimulation could enhance performance in specific tasks, such as maintaining safe driving distances or executing precise landings (Sansevere and Ward). However, the effects were highly context-dependent, influenced by factors such as participant expertise, task complexity, and the targeted brain region, again highlighting the need for further research to generalize findings across a broader range of real-world applications.

## Conclusion

Neurostimulation is a rapidly evolving field with significant implications for cognitive and perceptual enhancement. While studies have demonstrated its potential in improving attention, memory, decision-making, and learning, several factors—such as individual differences, task complexity, and ethical considerations—need to be addressed. Personalized neurostimulation protocols, methodological refinements, and regulatory frameworks will be crucial in ensuring the effective and responsible application of these technologies. As research advances, neurostimulation may become an integral tool for cognitive enhancement in both clinical and everyday settings, bridging the gap between neuroscience and real-world applications.
